# Supplementary material for: CCR4, a RNA decay factor, is hijacked by a plant cytorhabdovirus phosphoprotein to facilitate virus replication
Source: eLife. 2020 Mar 24;9:e53753. doi: 10.7554/eLife.53753 (PMC7105381; doi:10.7554/eLife.53753)
Supplement: Supplementary file 1. [file elife-53753-supp1.docx]

| Number | Accession | Candidates |
| --- | --- | --- |
| 1 | AK374808 | Carbon catabolite repression 4 |
| 2 | [YP_009649021.1](https://www.ncbi.nlm.nih.gov/protein/YP_009649021.1?report=genbank&log$=protalign&blast_rank=1&RID=788AWJXA01R) | ribulose-1,5-bisphosphate carboxylase/oxygenase large subunit |
| 3 | KAE8805896.1 | Elongation factor 1-alpha |
| 4 | YP_008757384 | ATP synthase subunit 1 |
| 5 | XP_020147764 | chlorophyll a-b binding protein |
| 6 | YP_007026453 | photosystem II CP43 chlorophyll apoprotein |
| 7 | AAA62325 | HSP70 |
| 8 | ABG75917 | vacuolar proton ATPase subunit |
| 9 | AHV84766 | histone 4 |
| 10 | KAE8813292 | Histone H2B |
| 11 | KAE8820295 | protoporphyrinogen oxidase |

**Supplementary File 1.** List of the BYSMV P protein interacting barley proteins obtained in IP-MS assays
