# Supplementary material for: CCR4, a RNA decay factor, is hijacked by a plant cytorhabdovirus phosphoprotein to facilitate virus replication
Source: eLife. 2020 Mar 24;9:e53753. doi: 10.7554/eLife.53753 (PMC7105381; doi:10.7554/eLife.53753)
Supplement: Supplementary file 2. [file elife-53753-supp2.docx]

**Supplementary File 2.** Primers used in this study.

| Primer | Sequence (5' to 3') | Notes |
| --- | --- | --- |
| BYS-RT-1 | ACGACCAGTGATCGTATAATTTGATTATTGGTGATC | For BYSMV gRNA RT primer |
| Oligo dT | GGATATCTGCAGGATCCAAGCTTTTTTTTTTTTTTTTTT | For RT primer |
| N-86-F | GAAGATCATGGATTGGACAGAGAG | To amplify N |
| N-216-R | GCAGGAGTGTAAACCGGGAT |  |
| P-137-F | AGATGGGATCTTCGGTGAGC | To amplify P |
| P-267-R | CTTCCACACCGGAGATATACC |  |
| HvEF1α-F | ACCTTTGCCACTTACCCTCCTC | To amplify EF1α of barley |
| HvEF1α-R | CCTTCTTCTCCACGCCCTTGAT |  |
| GDGm-HvCCR4-F | ctctctacaagatctcATGCTGAGTGTGGTACGGGT | For assembling pHvCCR4-GFP |
| GDGm-HvCCR4-R | gacaagcttgagctcTCGTCTGATTCTAGGCTTGCA |  |
| peHvCCR4-F | agaaggagatatacaATGCTGAGTGTGGTACGGGTGCAC | For assembling pHvCCR4-6×his |
| peHvCCR4-R | ggtggtggtgctcgagTCGTCTGATTCTAGGCTTGCAACG |  |
| OE-HvCCR4-F | aggatccccgggtacATGCTGAGTGTGGTACGGGTGCAC | For assembling pHvCCR4-3×flag |
| OE-HvCCR4-R | tgccacctccactagtTCGTCTGATTCTAGGCTTGCAACG |  |
| CCR4-N260A-F | CACAGTTCTGTCTTATGCTATTCTTGCAGATGCATATGCTAC | For Quickchange of HvCCR4_mEEP_ |
| CCR4-N260A-R | ATGCATCTGCAAGAATAGCATAAGACAGAACTGTGAATGTCCC |  |
| CCR4-E305A-F | TATCATTTGTCTTCAGGCTGTACAATTGAACCACTTCGAAGA |  |
| CCR4-E305A-R | TGGTTCAATTGTACAGCCTGAAGACAAATGATATCAGCATGATA |  |
| Bam-Hv.ccr4-F | gcctggcgcgccactagtggaATGCTGAGTGTGGTACGGGTGCAC | For assembling pNE and pCE |
| H1-Hv.ccr4-R | gtcgacagtactatcgatggaTCGTCTGATTCTAGGCTTGCAACG |  |
| YFPn-P_1-33_-F | ccaggcctactagtggaATGAGCTCATCCAATGCTGCA | For assembling P truncation vectors of pNE and pCE |
| YFPn -P_1-33_-R | tcgaggtcgacggatccTTACTCACCGAAGATCCCATCTAAA |  |
| YFPn-P_60-295_-F | ccaggcctactagtggaATGAAGGGGGAGAGGTATATCTCCGG |  |
| YFPn -P_60-295_-R | tcgaggtcgacggatccTTAGAGATCTCCATAAGGATCATGACGG |  |
| YFPn-P_183-295_-F | ccaggcctactagtggaATGAAGACTGTGGTAGAAAGTGCTT |  |
| YFPn –P_183-295_-R | tcgaggtcgacggatccTTAGAGATCTCCATAAGGATCATGACGG |  |
| YFPn-P_60-183_-F | ccaggcctactagtggaATGAAGGGGGAGAGGTATATCTCCGG |  |
| YFPn -P_60-183_-R | tcgaggtcgacggatccTTAAACCTCTGATACCATGGATTCCATCTTC |  |
| Bam-Hv.ccr4-C-F | gcctggcgcgccactagtggaTCTTTCGGGACATTCACAGTTCTG | For assembling truncation of pNE and pCE |
| H1-N-Hv.ccr4-R | gtcgacagtactatcgatggaAGAACTCTGGCTGTCCATATCCAAGT |  |
| qNbEF1α-F | AGCTTTACCTCCCAAGTCATC | To amplify EF1α of tabacco |
| qNbEF1α-R | AGAACGCCTGTCAATCTTGG |  |
| OE-HvCAF1-F | aggatccccgggtacATGGCGATGTCGGATCTCGCGG | For assembling pHvCAF1-3×flag |
| OE-HvCAF1-R | tgccacctccactagtATGCGCACTGACACCATTCTCTGC |  |
| Super-Hv.Ccr4-F | tctgcagggcccggggtATGCTGAGTGTGGTACGGGTGCAC | For assembling pHvCCR4-mCherry |
| Super-Hv.Ccr4-R | agtatttaaatgtcgaccTCGTCTGATTCTAGGCTTGCAACG |  |
| N-Kpn1-F | GGGGTACCATGGCAAAAGAAGATCATGGA | For construct of pMDC32-1×flag by kpn1 and Pac1 |
| N-Pac1-R | CCTTAATTAATTACTTATCGTCGTCATCCTTGTAATCGGAGAAGATCTGGTCAGCATT |  |
| P-6myc-F | cttcgaattctgcagtcATGAGCTCATCCAATGCTGCA | For assembling pP-6×myc |
| P-6myc-R | ccgcggtaccgtcgacGAGATCTCCATAAGGATCATGACG |  |
| HvCCR4-6myc-F | cttcgaattctgcagtcATGCTGAGTGTGGTACGGGTGCAC | For assembling pHvCCR4-6×myc |
| HvCCR4-6myc-R | ccgcggtaccgtcgacTCGTCTGATTCTAGGCTTGCAACG |  |
| GEX-P-F | ctggttccgcgtggaATGAGCTCATCCAATGCTGCA | For assembling pGST-P |
| GEX-P-R | cggaaattcccggggaGAGATCTCCATAAGGATCATGACG |  |
| trailer-F | GTCTAATAAGCGATGCGTAA | To amplify trailer of gRNA |
| trailer-R | CTACCTCTCACACACTCTT |  |
| rtRFP-F | CCTGGTGGAGTTCAAGTC | To amplify RFP |
| rtRFP-R | CACGATGGTGTAGTCCTC |  |
| BY-Hv.Ccr4-F | caaatcttggtcaataATGCTGAGTGTGGTACGGGTGCAC | For assembling pCB-BY-HvCCR4 |
| BY-Hv.Ccr4-R | cccgattcctacgcgTCATCGTCTGATTCTAGGCTTGCAA |  |
| BY-Hv.Ccr4fs-F | caaatcttggtcaataATGTCTGAGTGTGGTACGGGTGCAC |  |
| LstCCR4-F | ATGCTCTCAAAAGCTTACACCG | To amplify LstCCR4 |
| LstCCR4-R | CCTTCGAGGAATGAGACCGTTG |  |
| T7- LstCCR4-14-F | TAATACGACTCACTATAGGGCTTACACCGATGAAGAAGATGC | For transcription of dsRNA |
| T7- LstCCR4-438-R | TAATACGACTCACTATAGGGCAATTTACCGATTTCGTAGGGC |  |
| LstCCR4-1009-F | GACATGCTGAACAGAGTCATGC | For Real-time detection |
| LstCCR4-1205-R | CACAGCATCATCACCTGGATCA |  |
| GEX-LstCCR4-F | ctggttccgcgtgga ATGCTCTCAAAAGCTTACACCG | For assembling pGST-LstCCR4 |
| GEX-LstCCR4-R | cggaaattcccgggga CTACCTTCGAGGAATGAGACCGT |  |
| YFP-NCMV-P-F | ccaggcctactagtgga ATGGATAAGAAAGCAAGTGGAATC | For assembling pNE and pCE |
| YFP-NCMV-P-R | tcgaggtcgacggatcc TTAAAAGTCGGCATACGGGTC |  |

/F: forward primer; /R: reverse primer. The sequence shown in lower letters is homologous to other primer as indicated in Notes column, to facilitate In-Fusion cloning;
