# Supplementary material for: CCR4, a RNA decay factor, is hijacked by a plant cytorhabdovirus phosphoprotein to facilitate virus replication
Source: eLife. 2020 Mar 24;9:e53753. doi: 10.7554/eLife.53753 (PMC7105381; doi:10.7554/eLife.53753)
Supplement: Supplementary file 3. [file elife-53753-supp3.docx]

**Supplementary File 3.** **Key Resources Table**

| Key Resources Table | | | | |
| --- | --- | --- | --- | --- |
| Reagent type (species) or resource | Designation | Source or reference | Identifiers | Additional information |
| strain, strain background (*Escherichia coli*) | *E. coli* BL21(DE3) | Thermo  Fisher  Scientific | C601003 | Prokaryotic expression protein |
| strain, strain background (*Escherichia coli*) | *E. coli* DH5α  (DE3) | Thermo  Fisher  Scientific | 18258012 | Clone vectors |
| antibody | Anti-Flag  (Mouse monoclonal) | Sigma | F1804-200UG | WB (1:5000) |
| antibody | Anti-His  (Mouse monoclonal) | proteintech | Cat No: 66005-l-lg | WB (1:5000) |
| antibody | Anti-GST  (Rabbit polyclonal) | Thermo  Fisher  Scientific | A-5800 | WB (1:10000) |
| antibody | Anti-RFP  (Rabbit polyclonal) | This paper |  | WB (1:3000)  (Materials and methods section) |
| antibody | Anti-P  (Rabbit polyclonal) | This paper |  | WB (1:3000)  (Materials and methods section) |
| antibody | Anti-N  (Rabbit polyclonal) | This paper |  | WB (1:3000)  (Materials and methods section) |
| commercial assay or kit | SE Seamless cloning and Assembly Kit | ZOMANBIO | ZC231-2 |  |
| commercial assay or kit | HiScript II Reverse Transcriptase | Vazyme | Vazyme Code: R201-02 |  |
| commercial assay or kit | T7 RiboMAX^TM^ Express RNAi System | Promega | P1700 |  |
| commercial assay or kit | 2×Phanta Max Master Mix | Vazyme | Vazyme Code: P515-02 |  |
| commercial assay or kit | SsoFast EvaGreen Supermix | Bio-Rad | Cat.#1725201 |  |
| commercial assay or kit | TIANprep Rapid Mini Plasmid Kit | TIANGEN | Cat.#DP105-03 |  |
| recombinant DNA reagent | pET30a-HvCCR4 | This paper |  | For purification of HvCCR4-his  (Materials and methods section) |
| recombinant DNA reagent | pET30a-P | This paper |  | For purification of P-his (Materials and methods section) |
| recombinant DNA reagent | pET30a-N | This paper |  | For purification of N-his (Materials and methods section) |
| recombinant DNA reagent | pGEX-P | This paper |  | For purification of GST-P (Materials and methods section) |
| recombinant DNA reagent | pGDGm-P | This paper |  | For expression of P-GFP (Materials and methods section) |
| recombinant DNA reagent | pMDC32-HvCCR4 | This paper |  | For expression of HvCCR4-3Flag (Materials and methods section) |
| recombinant DNA reagent | pMDC32-HvCCR4^N^ | This paper |  | For expression of HvCCR4^N^-3Flag (Materials and methods section) |
| recombinant DNA reagent | pMDC32-HvCCR4^C^ | This paper |  | For expression of HvCCR4^C^-3Flag (Materials and methods section) |
| recombinant DNA reagent | pMDC32-HvCCR4^mEEP^ | This paper |  | For expression of HvCCR4^mEEP^-3Flag (Materials and methods section) |
| recombinant DNA reagent | pMDC32-HvCAF1 | This paper |  | For expression of HvCAF1-3Flag (Materials and methods section) |
| recombinant DNA reagent | pY^N^-P | This paper |  | For expression of Y^N^-P (Materials and methods section) |
| recombinant DNA reagent | pY^C^-P | This paper |  | For expression of Y^C^-P (Materials and methods section) |
| recombinant DNA reagent | pY^N^-HvCCR4 | This paper |  | For expression of Y^N^-HvCCR4 (Materials and methods section) |
| recombinant DNA reagent | pY^N^-HvCCR4^N^ | This paper |  | For expression of Y^N^-HvCCR4^N^ (Materials and methods section) |
| recombinant DNA reagent | pY^N^-HvCCR4^C^ | This paper |  | For expression of Y^N^-HvCCR4^C^ (Materials and methods section) |
| recombinant DNA reagent | pY^C^-HvCCR4 | This paper |  | For expression of Y^C^-HvCCR4 (Materials and methods section) |
| recombinant DNA reagent | pY^N^-NCMV-P | This paper |  | For expression of Y^N^-NCMV-P (Materials and methods section) |
| recombinant DNA reagent | pY^C^-NCMV-P | This paper |  | For expression of Y^C^-NCMV-P (Materials and methods section) |
| recombinant DNA reagent | pY^C^-HvCAF1 | This paper |  | For expression of Y^C^-HvCAF1 (Materials and methods section) |
| recombinant DNA reagent | pY^C^-lsCCR4 | This paper |  | For expression of Y^C^-lsCCR4  (Materials and methods section) |
| recombinant DNA reagent | pY^C^-lsCCR4^N^ | This paper |  | For expression of Y^C^-lsCCR4^N^  (Materials and methods section) |
| recombinant DNA reagent | pY^C^-lsCCR4^C^ | This paper |  | For expression of Y^C^-lsCCR4^C^  (Materials and methods section) |
| recombinant DNA reagent | pGDG-P1-207 | This paper |  | For expression of GFP-P1-207 |
| recombinant DNA reagent | pGDGm-HvCCR4 | This paper |  | For expression of HvCCR4-GFP (Materials and methods section) |
| recombinant DNA reagent | pSuper-HvCCR4 | This paper |  | For expression of HvCCR4-mCherry (Materials and methods section) |
| recombinant DNA reagent | pGDGc-P | This paper |  | For expression of CFP-P |
| recombinant DNA reagent | pMAL-HvCAF1 | This paper |  | For purification of MBP-HvCAF1 (Materials and methods section) |
| recombinant DNA reagent | pBYR-GUS | This paper |  | For expression of cDNA clone of BY-GUS-RFP (Materials and methods section) |
| recombinant DNA reagent | pBYR-HvCCR4 | This paper |  | For expression of cDNA clone of BY-HvCCR4-RFP (Materials and methods section) |
| recombinant DNA reagent | pBYR-HvCCR4^mEEP^ | This paper |  | For expression of cDNA clone of BY-HvCCR4^mEEP^-RFP (Materials and methods section) |
| software, algorithm | ImageJ | NIH |  | Quantify gels bands |
| software, algorithm | GraphPad Prism | GraphPad |  | Statistic datas |
| software, algorithm | LAS X | Leica Application suit X |  | Confocal observation |
